# *SIX1* branchio-oto-renal syndrome variants have different effects on embryonic craniofacial gene expression and cartilage formation

**DOI:** 10.1242/dev.205428

**Published:** 2026-07-08

**Authors:** Kelsey Coppenrath, Nikko-Ideen Shaidani, Thomas Naert, Himani D. Majumdar, Marko Horb, Soeren S. Lienkamp, Steven L. Klein, Sally A. Moody

**Affiliations:** ^1^ Eugene Bell Center for Regenerative Biology and Tissue Engineering and National Xenopus Resource, Marine Biological Laboratory, Woods Hole, MA 02543, USA; ^2^ Institute of Anatomy, University of Zurich, 8057 Zurich, Switzerland; ^3^ Department of Biomedical Molecular Biology, Ghent University, B-9000 Ghent, Belgium; ^4^ Department of Anatomy & Cell Biology, George Washington University School of Medicine and Health Sciences, Washington, DC 20037, USA; ^5^ Department of Health Sciences and Technology, ETH Zurich, 8092 Zurich, Switzerland

**Keywords:** Otic vesicle, Neural crest, Preplacodal ectoderm, Cranial cartilage, Branchio-oto-renal syndrome, Eya1

## Abstract

*SIX1* variants underlying branchio-oto-renal syndrome occur in the SIX domain (SD) or homeodomain (HD). We tested whether different variants – V17E (SD), Y129C (HD) – cause distinct developmental phenotypes in *Xenopus* embryos with reduced Six1 in comparison to wild-type Six1 (Six1WT). In Six1 morphants, Six1WT restored neural crest and preplacodal gene expression; V17E restored *foxd3* and *irx1* better than Y129C, and Y129C restored *sox11* better than V17E. In *six1*-null otic vesicles, Six1WT partially restored *tbx1* and *sobp*, V17E was less effective and Y129C was least effective; all three restored *dlx5*. In *six1* heterozygotes, Six1WT and Y129C had similar pleiotropic effects on *tbx1*, whereas V17E had no effect; Six1WT restored *dlx5* expression, V17E was less effective and Y129C was most deficient*.* In *six1-*null tadpoles, reduced cranial cartilage volume and individual cartilage abnormalities were rescued by Six1WT, less so by V17E and not by Y129C. In heterozygotes and wild types, Y129C caused a higher frequency of abnormal cartilages compared to Six1WT or V17E. Thus, variants with different functional deficits have distinguishable effects in both nulls and heterozygotes on the formation of the tissues affected in branchio-oto-renal syndrome.

## Teaser

**Summary:** Expression of branchio-oto-renal syndrome-associated *SIX1* variants in Six1-depleted *Xenopus* embryos differentially affects neural crest, preplacodal ectoderm and otic vesicle gene expression, and cranial cartilage volume and morphology.

## INTRODUCTION

Branchio-oto-renal syndrome (BOR) is an autosomal dominant hearing loss syndrome. Affected individuals present with highly variable combinations of malformations in neural crest (NC)-derived structures (hyoid region, external ear, middle ear), cranial placode-derived structures (inner ear) and in some cases kidney, and variants in *SIX1* and *EYA1* are causative in about half of affected individuals (Neal et al., 2024; Smith and Azaiez, 2025). Individuals diagnosed as branchio-otic syndrome 3 (BOS3; OMIM #608389) carry single-nucleotide missense mutations or deletions in the gene encoding the SIX1 transcription factor, activity of which is modulated by co-factors such as EYA1 (Ikeda et al., 2002; Li et al., 2003; Silver et al., 2003; Tavares et al., 2021; Neal et al., 2024).

**Fig. 1.**
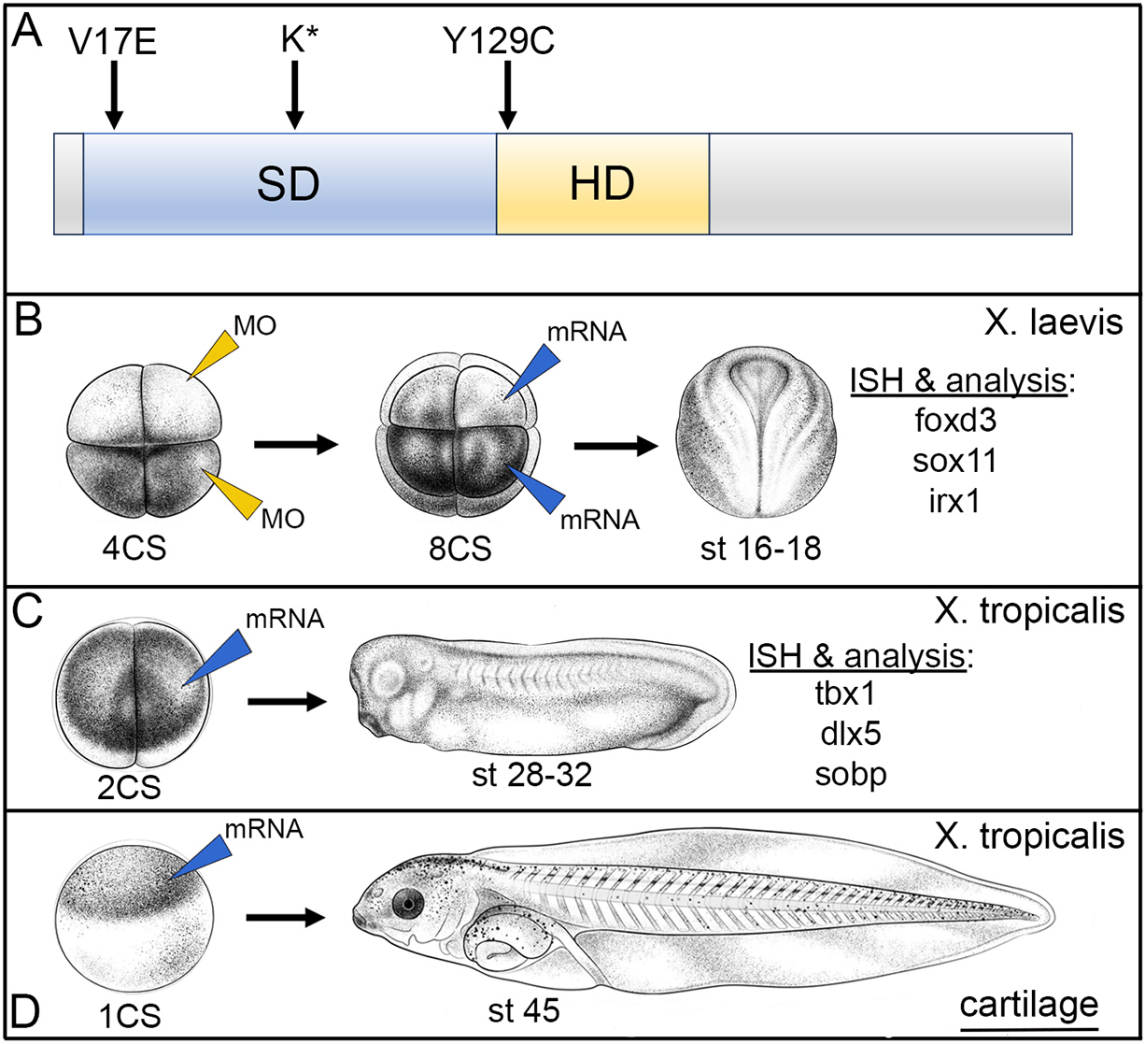
**Six1 protein domains and experimental approaches.** (A) *Xenopus* Six1 protein indicating the SIX domain (SD), homeodomain (HD) and two BOR variants (V17E, Y129C). K* indicates the stop codon in *X. tropicalis* mutants. (B) At the 4-cell stage (4CS), the animal poles of the left blastomeres of *X. laevis* embryos were injected with translation-blocking Six1 morpholinos (MO). At the next cell cycle (8CS), the animal pole daughters were injected with mRNA encoding either wild-type or variant Six1. At neural plate stages, embryos were fixed and processed for NC and PPE gene expression by ISH. (C) At the 2-cell stage (2CS), one blastomere of *X. tropicalis* embryos from the *six1*-mutant line was injected with mRNAs. At stages 28-32, larvae were fixed, genotyped and processed for OV gene expression by ISH. (D) At the 1-cell stage (1CS), *X. tropicalis* zygotes from the *six1*-mutant line were injected with mRNAs. Tadpoles were fixed, genotyped and processed for changes in cartilage volume or morphology. *Xenopus* illustrations © Natalya Zahn (2022) (Xenbase; www.xenbase.org; RRID:SCR_003280; Zahn et al., 2022). Illustrations are published under the terms of a CC BY-NC 4.0 license.

Studies of the developmental function of Six1 demonstrate that it plays a central role in the formation of structures derived from cranial NC and preplacodal ectoderm (PPE). Six1 loss of function in *Xenopus*, zebrafish, chick and mouse results in reduced expression of several PPE genes and defects in otic and craniofacial development (Laclef et al., 2003; Zheng et al., 2003; Brugmann et al., 2004; Ozaki et al., 2004; Zou et al., 2004; Bricaud and Collazo, 2006; Konishi et al., 2006; Chen et al., 2009; Christophorou et al., 2009; Ikeda et al., 2010; Coppenrath et al., 2021). Six1 contains an N-terminal protein–protein interaction domain (SD) and a homeodomain (HD) (Pignoni et al., 1997; Kawakami et al., 2000). The majority of causative *SIX1* BOR variants are missense mutations that result in single amino acid substitutions or deletions in either the SD or HD (Fig. 1A; Neal et al., 2024). In cultured mammalian cells, V17E (SD) abolishes the SIX1–EYA1 interaction and prevents the translocation of EYA1 into the nucleus and Y129C (HD) significantly reduces DNA binding and transcription via a luciferase reporter construct (Ruf et al., 2004; Sanggaard et al., 2007; Kochhar et al., 2008; Patrick et al., 2009, 2013; Shah et al., 2020; Lee et al., 2023).

Previously, we found that V17E and Y129C have different effects on gene expression when overexpressed in wild-type *Xenopus* embryos that carry a normal endogenous level of Six1 (Shah et al., 2020; Mehdizadeh et al., 2021). In these embryos, V17E altered the expression of NC, PPE and otic vesicle (OV) genes at frequencies very similar to an equivalent dose of wild-type Six1, whereas Y129C had notably weaker effects. To make those findings more relevant to the heterozygous BOR genotype, herein we expressed the variants on reduced Six1 backgrounds and assessed how they altered the expression of genes that are required for the formation of the NC, PPE and OV, embryonic precursor populations highly relevant to BOR phenotypes. We also assessed the formation of tadpole cranial cartilage, derived from cranial NC, by quantifying cartilage volume using a deep learning-based 3D reconstruction approach (Naert et al., 2021), and analyzing dysmorphologies in individual cartilages. These analyses demonstrate that variants with different functional deficits have distinguishable effects in both nulls and heterozygotes that impact the early development and morphogenesis of tissues affected in BOR.

## RESULTS

### BOR variants differentially alter NC and preplacodal gene expression

Individuals with BOR carry one copy of wild-type *SIX1* and one copy of a causative variant. To approximate a heterozygous level of endogenous Six1 protein on one side of wild-type *Xenopus laevis* embryos, Six1 translation-blocking morpholino oligonucleotides (MOs) were injected into the animal pole of the blastomeres on the left side of the 4-cell embryo (Fig. 1B). These MOs were previously demonstrated to be specific and efficiently knockdown translation of endogenous protein (Brugmann et al., 2004; Sullivan et al., 2019). At neural plate stages, embryos were processed by *in situ* hybridization (ISH) for the expression of genes involved in the formation of the NC and PPE (Fig. 1B), precursor populations of the cranial cartilages and inner ear that are dysmorphic in BOR. *foxd3* is essential for determining the cranial NC (Dottori et al., 2001; Sasai et al., 2001), *irx1* is required for the formation of both NC and PPE (Glavic et al., 2004), and *sox11* is required for PPE formation and neurogenesis (Brugmann et al., 2004; Chen et al., 2016). In each MO-injected embryo, the intensity of ISH staining was compared between injected (left) and uninjected (right) sides and qualitatively scored in categories: ‘fainter’, ‘larger’ or ‘same’ (Table 1; see Materials and Methods for details). Bilateral comparison within the same embryo is essential because there is up to a 38% variation in diameter and 2.6-fold difference in volume in *Xenopus* zygotes, even in clutches derived from the same female (Leibovich et al., 2020). These size differences, as well as differences in the rates at which siblings grow, result in wide variations in gene expression levels and domain sizes between embryos, making intra-embryo comparisons essential.

**Table 1.** Percentages of embryos with the noted intensity of ISH staining on the injected side compared to the uninjected side of the same embryo

	MO-only	MO+Six1WT	MO+V17E	MO+Y129C
*foxd3*	(37)	(42)	(45)	(42)
Fainter	2.7	**83.3**	**73.3**	**23.9**
Larger	**78.4**	2.40	15.6	38.1
Same	18.9	14.3	11.1	38.0
*irx1*	(47)	(40)	(41)	(78)
Fainter	0	**82.5**	**80.5**	**59.0**
Larger	**66.0**	0	4.9	11.5
Same	34.0	17.5	14.6	29.5
*sox11*	(34)	(33)	(44)	(48)
Fainter	**82.4**	36.4	68.2	10.4
Larger	2.9	27.3	20.5	2.1
Same	14.7	36.4	11.4	87.5
Larger+same	17.6	**63.7**	**31.9**	**89.6**

Differences were scored as fainter, larger or the same (see Materials and Methods). Bold indicates effects of: (1) MO-only; (2) Six1WT differences from MO-only; and (3) corresponding effects caused by the variants. Numbers in parentheses indicate the number of embryos examined.

**Fig. 2.**
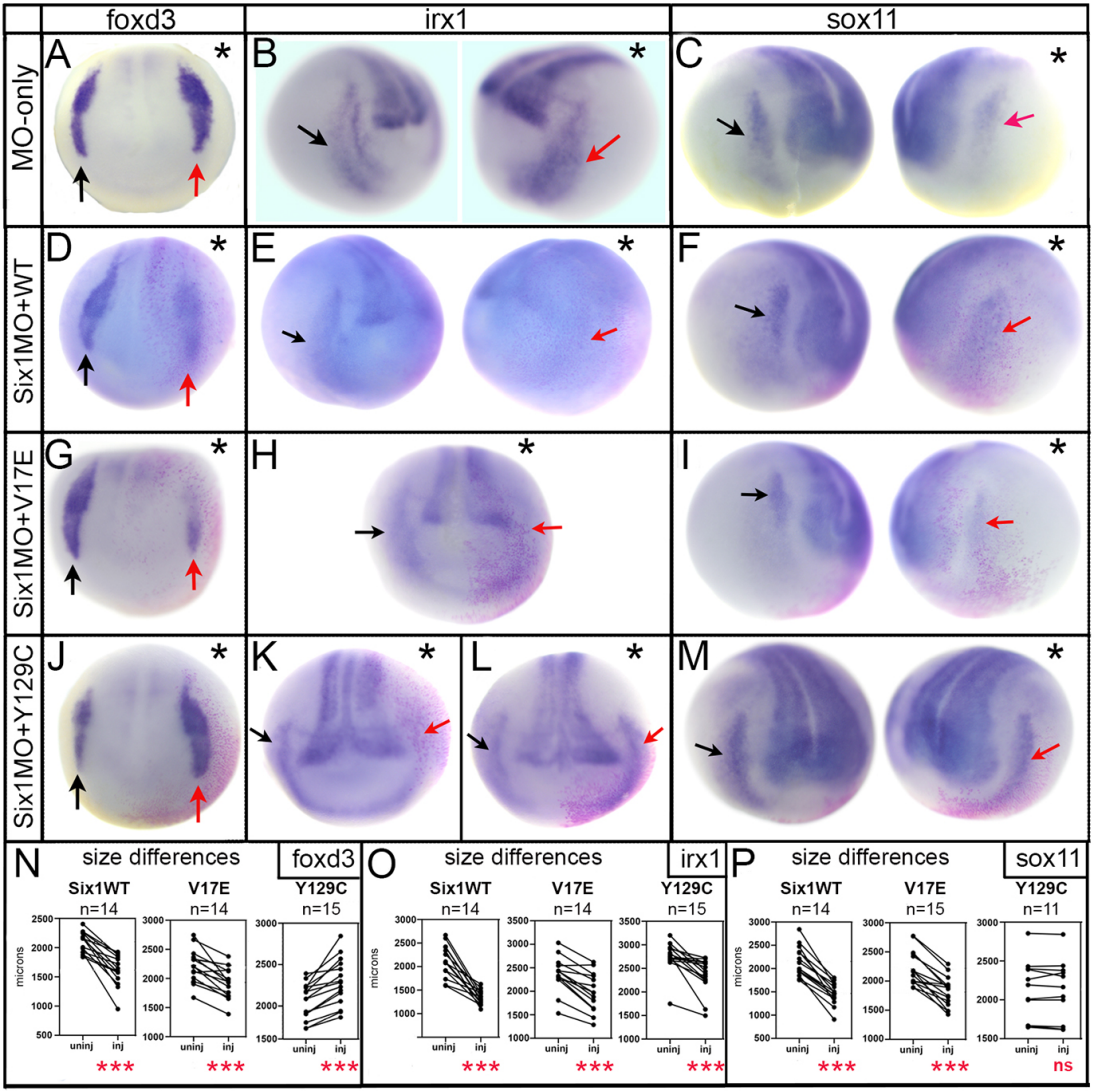
**BOR variants differentially affect NC and PPE expression domains.** (A-C) Morphants injected only with Six1MO (MO-only) on the left side and processed by ISH for *foxd3* (A), *irx1* (B) or *sox11* (C). (D-F) Examples of morphants injected with wild-type *six1* mRNA (Six1MO+WT) that have fainter staining of *foxd3* (D), *irx1* (E) and *sox11* (F) on the injected compared to uninjected sides. (G-I) Examples of morphants injected with V17E mRNA (Six1MO+V17E) that have fainter staining of *foxd3* (G), *irx1* (H) and *sox11* (I) on the injected side. (J-M) Examples of morphants injected with Y129C mRNA (Six1MO+Y129C) that have a larger *foxd3* domain (J), a fainter (K) or larger (L) *irx1* domain and the same *sox11* domain (M) on the injected side. In A-M, asterisks denote injected morphant side, black arrows denote expression domains on uninjected side, and red arrows denote expression domains on morphant side of the same embryo. (N) The perimeter of the *foxd3* NC domain was measured in a subset of embryos. For Six1WT- and V17E-injected morphants with fainter staining on the injected side (inj), the domains were significantly smaller compared to uninjected side (uninj) of the same embryo. For Y129C-injected morphants, those visually scored with a larger staining domain measured significantly larger. ***P<0.0001. (O) For all three proteins, the size of the fainter *irx1 PPE* domain was significantly smaller on the injected side. ***P<0.0001. (P) For Six1WT and V17E, the *sox11* domain was significantly smaller on the injected side. ***P<0.0001. For Y129C, it was the same on both sides. ns, not significant (*P*>0.05).

The majority of MO-injected embryos (MO-only) had larger *foxd3* NC domains*,* larger *irx1* PPE domains and fainter *sox11* PPE domains on the injected morphant side (Fig. 2A-C; Table 1), indicating that Six1 normally restricts *foxd3* NC and *irx1* PPE domains, and expands *sox11* PPE domains. To determine the ability of wild-type Six1 to rescue the effect of reduced endogenous protein, we injected its mRNA (Six1WT) into the two 8-cell daughters of the MO-injected blastomere precursors of the NC and PPE (Fig. 1B). Supplying Six1WT to the morphant side caused *foxd3* staining to be fainter on the injected side in the majority of embryos (Fig. 2D; Table 1), in significant contrast to MO-only (*P*<0.0001). Thus, Six1WT reversed the effects of endogenous Six1 knockdown. To confirm the staining categories, we also measured the sizes of *foxd3* domains on both sides of a subset of embryos scored as fainter (Fig. S1A); they were significantly smaller on the Six1WT-injected side (Fig. 2N), consistent with the scoring categories.

Supplying Six1WT to morphants also reduced the effects of MO injection on genes expressed in the PPE. In significant contrast to MO-only embryos (Fig. 2B), more Six1WT-injected morphants had fainter *irx1* PPE staining and none had larger domains (Fig. 2E; Table 1; *P*<0.0001). Supporting this scoring, the ‘fainter’ *irx1* domains were significantly smaller on the Six1WT-injected side (Fig. 2O). Although some Six1WT-injected morphants also had fainter *sox11* domains (Fig. 2F), compared to MO-only proportionally more had either larger domains or the same staining intensity (Table 1; *P*=0.0003). Measuring the *sox11* domains confirmed these qualitative categories (Fig. 2P; Fig. S1C,D). Thus, Six1WT partially reversed the effects of endogenous Six1 knockdown on both PPE genes.

To determine whether BOR variants restored gene expression as effectively as Six1WT, we injected their mRNAs into the daughters of the MO-injected blastomeres (Fig. 1B). For *foxd3*, most V17E-injected morphants had fainter staining and significantly smaller domains on the injected side (Fig. 2G,N), similar to Six1WT-injected morphants (*P*=0.1182; Table 1). Since V17E and Six1WT each caused smaller *foxd3* domains, we assessed whether the reductions were of the same magnitude by converting the size of the domain on the injected side to a percentage of that on the uninjected side of the same embryo; this normalizes inter-embryo size variation. The mean percentage reduction of *foxd3* domains induced by V17E injection (13.2%) was significantly less than that induced by Six1WT (23.1%; *P*=0.0073), indicating that V17E is less effective than Six1WT at correcting MO-caused changes. In comparison, only a small percentage of Y129C-injected morphants had fainter *foxd3* staining and many more had ‘larger’ or ‘same’ domains (Fig. 2J; Table 1), in proportions that significantly differed from Six1WT (*P*<0.0001) and V17E (*P*=0.004). Measuring the domains scored as larger (Fig. S1B) showed they were significantly larger on the Y129C-injected side (Fig. 2N), supporting the scoring categories and revealing that Y129C was less effective at restricting *foxd3* expression compared to Six1WT or V17E.

The majority of V17E-injected morphants had fainter *irx1* staining on the injected side (Fig. 2H; Table 1), similar to Six1WT (*P*=0.6076), indicating that it also restricted *irx1* expression. In comparison to Six1WT, Y129C-injected morphants had a smaller proportion of fainter *irx1* staining (Fig. 2K) and higher proportion of ‘larger’ and ‘same’ domains (Fig. 2L; Table 1; *P*=0.0113), indicating it is less effective than Six1WT at restricting *irx1* expression. Interestingly, its effects did not reach significance when compared to V17E (*P*=0.0621). For Six1WT, V17E and Y129C, the *irx1* domains scored as fainter also were significantly smaller (Fig. 2O), consistent with the scoring categories. The mean percentage differences in size for each variant (V17E, 16.2%; Y129C, 14.0%) was significantly smaller compared to Six1WT (35.2%; *P*<0.0001 each), but were similar to each other (*P*=0.5443). These results demonstrate that neither variant was as effective as Six1WT in restricting *irx1* expression.

Most V17E-injected morphants had fainter *sox11* staining on the injected side (Fig. 2I; Table 1), in proportions similar to MO-only (*P*>0.05) and significantly different from Six1WT (*P*<0.0001). The fainter *sox11* domains also were significantly smaller on the injected side (Fig. 2P), and the mean percentage reduction in domain size was significantly less for V17E (18.0%) compared to Six1WT (31.6%; *P*=0.0003). These data indicate that V17E was less effective at altering *sox11* expression than Six1WT in morphants. In contrast, in the majority of Y129C-injected morphants *sox11* staining was restored to the same staining intensity as the uninjected side (Fig. 2M; Table 1), a significant difference compared to Six1WT and V17E (*P*<0.0001 each). Consistent with the scoring categories, the sizes of the *sox11* domains on the injected and uninjected sides of Y129C-injected morphants were indistinguishable (Fig. 2P; *P*=0.4648).

In summary, these experiments reveal that in morphants Six1WT can partially restore the effects of reduced endogenous Six1 on NC and PPE genes. V17E was similar to Six1WT in that it partially restored *foxd3* and *irx1* domains, but was considerably less effective; Y129C was even less able to restore their expression. In contrast, V17E only weakly restored *sox11* expression, whereas Y129C was significantly more effective, even compared to Six1WT.

### BOR variants differentially alter OV gene expression

**Fig. 3.**
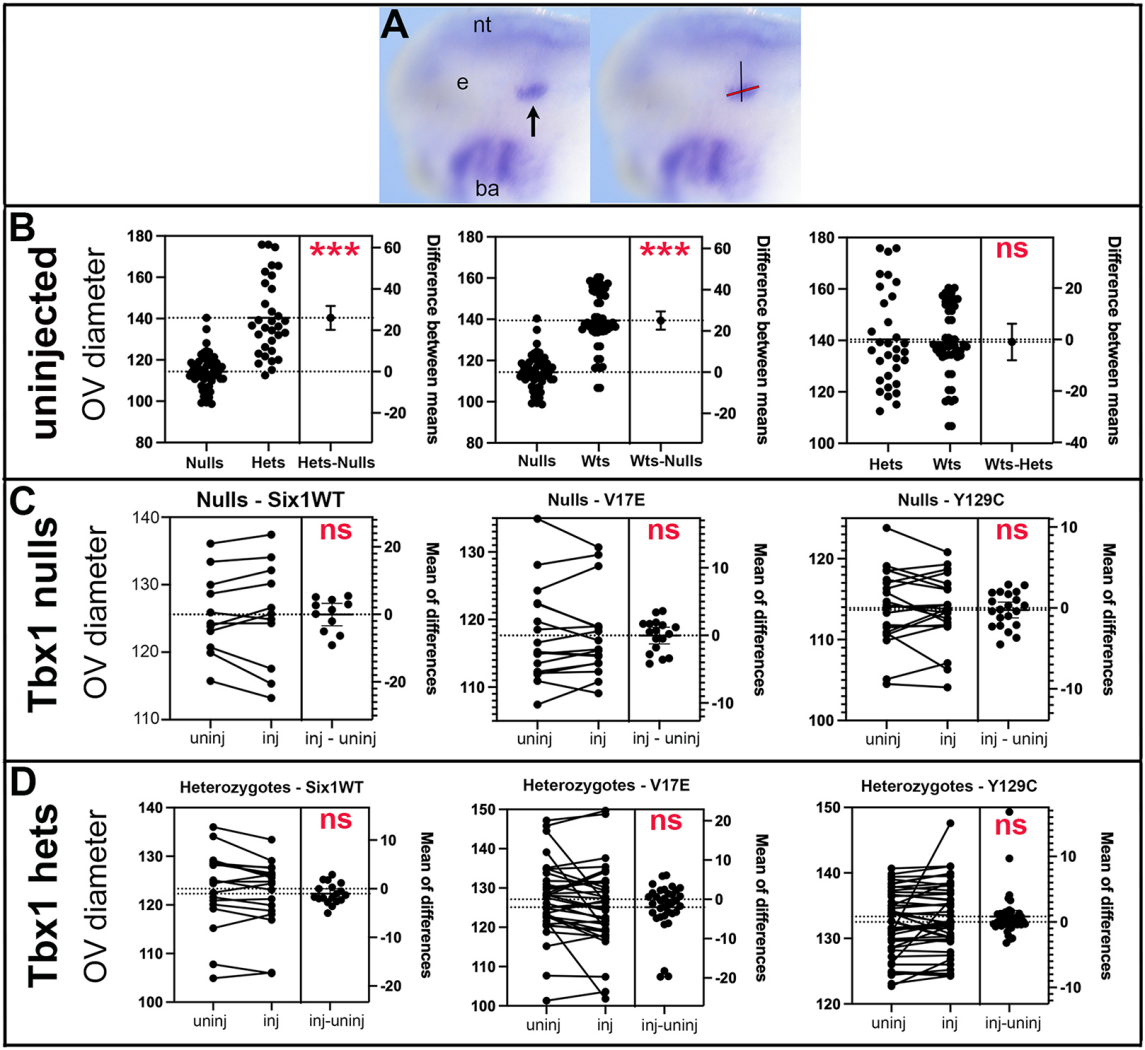
**Measurements of OV diameters in *six1-*nulls and heterozygotes.** (A) Left: *tbx1* OV expression (arrow) in a wild-type *X. tropicalis* larva. ba, branchial arches; e, eye; nt, neural tube. Right: The same image showing the length of the OV dorsal-ventral axis (black line) and the length of the *tbx1* OV domain (red line). (B) OV diameters (in μm) in the dorsal-ventral axis of uninjected nulls, heterozygotes (hets) and wild types (Wts) compared by unpaired *t*-test. ***P<0.0001. (C) OV diameters in *six1-*nulls processed for *tbx1* expression and injected with the indicated mRNAs did not show significant differences between the uninjected (uninj) and injected (inj) sides of the same embryo. (D) OV diameters in *six1* heterozygotes processed for *tbx1* expression and injected with the indicated mRNAs did not show significant differences between the uninjected and injected sides of the same embryo. ns, not significant. Error bars represent s.d. Dotted lines indicate 95% confidence interval.

Since BOR variants differentially affected PPE gene expression, we examined whether they also have different effects on OV development. We addressed this question in a *Xenopus tropicalis* line that carries genetically reduced levels of Six1; this line was not used for the experiments described above due to difficulties in dissecting sufficient material for genotyping at neural plate stages. We first assessed whether the size of the OV was different between genotypes by measuring its diameter in the dorsal-ventral axis (Fig. 3A). The *six1*-null OVs were significantly smaller compared to heterozygotes or wild types (Fig. 3B), similar to observations in mouse (Laclef et al., 2003; Zou et al., 2004). Next, we tested whether Six1WT or the variants could restore the effects of reduced Six1 by injecting their mRNAs on one side at the 2-cell stage and examining larvae for the expression of three genes important for OV development: *tbx1*, *dlx5*, *sobp* (Raft et al., 2004; Merlo et al., 2002; Tavares et al., 2021) (Fig. 1C). Note that although Six1WT and variant mRNAs were synthesized from *X. laevis* plasmids, the *X. tropicalis* protein is identical in the SD and HD, the functional domains affected in BOR (Fig. S2).

**Fig. 4.**
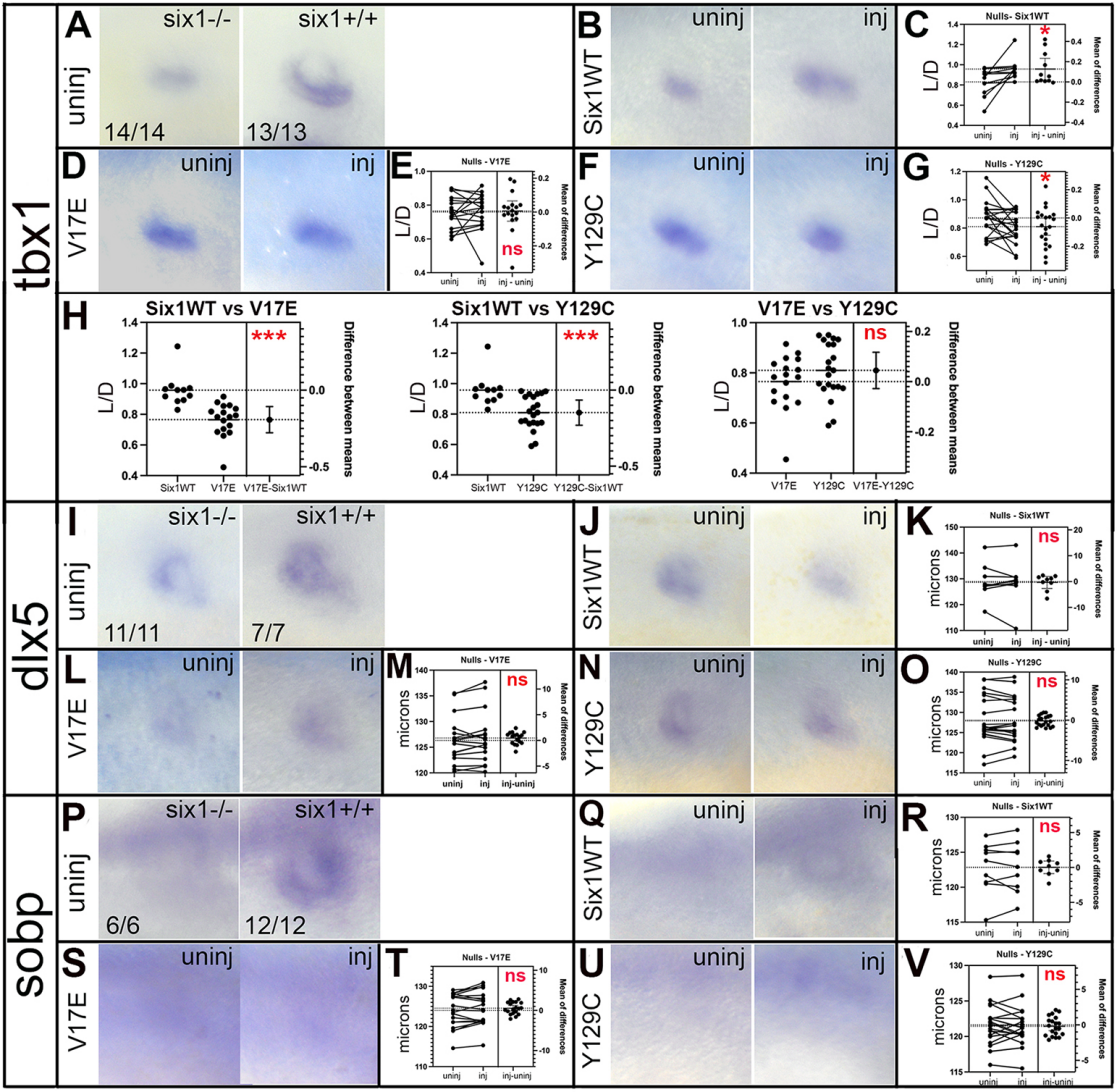
**In *six1*-nulls, BOR variants differentially alter OV gene expression.** (A) *tbx1* expression was reduced in uninjected *six1^−/−^* compared to *six1^+/+^*. The number of larvae showing the indicated staining pattern is indicated. (B) Most Six1WT-injected *six1*-nulls had a visually larger *tbx1* domain on the injected (inj) compared to uninjected (uninj) side of the same embryo. (C) The lengths of the *tbx1* domain/OV diameter (L/D) on each side of Six1WT-injected *six1*-nulls were significantly larger. *P<0.05. (D) In the majority of V17E-injected *six1*-nulls, there was no visual change in *tbx1* OV staining on the injected side. (E) The L/Ds on each side of V17E-injected *six1*-nulls were not significantly different. (F) In the majority of Y129C-injected *six1*-nulls, the *tbx1* OV staining was visually smaller on the injected side. (G) The L/Ds on each side of Y129C-injected *six1*-nulls were significantly smaller. *P<0.05. (H) The LDs of Six1WT were significantly different from each variant (***P<0.001). The LDs of the variants were not significantly different from each other. (I) *dlx5* OV expression was fainter in uninjected *six1*-nulls compared to wild types. (J) Most Six1WT-injected *six1*-nulls had a fainter *dlx5* domain on the injected side. (K) The OV diameter was not significantly different between injected and uninjected sides of Six1WT-injected *six1*-nulls stained for *dlx5*. (L) In some V17E-injected *six1*-nulls, *dlx5* OV staining was darker on the injected side. (M) The OV diameter was not significantly different between injected and uninjected sides of V17E-injected *six1*-nulls stained for *dlx5*. (N) In some Y129C-injected *six1*-nulls, *dlx5* OV staining was the same on both the injected and uninjected sides. (O) The OV diameter was not significantly different between injected and uninjected sides of Y129C-injected *six1*-nulls stained for *dlx5*. (P) *sobp* OV staining was not detected in uninjected *six1*-nulls, but strong in wild types. (Q) Many Six1WT-injected *six1*-nulls had darker *sobp* staining on the injected side. (R) The OV diameter was not significantly different between injected and uninjected sides of Six1WT-injected *six1*-nulls stained for *sobp*. (S) In most V17E-injected *six1*-nulls, *sobp* staining was not detected on the injected side. (T) The OV diameter was not significantly different between injected and uninjected sides of V17E-injected *six1*-nulls stained for *sobp*. (U) *sobp* staining was not detected in any Y129C-injected *six1*-nulls. (V) The OV diameter was not significantly different between injected and uninjected sides of Y129C-injected *six1*-nulls stained for *sobp*. ns, not significant. Error bars represent s.d. Dotted lines indicate 95% confidence interval.

Tbx1 was expressed in *six1*-null OVs, but the domain was visually fainter or smaller compared to wild type (Fig. 4A). Because a smaller domain might simply result from a smaller OV diameter, we first compared the OV diameters on both sides of Six1WT-injected *six1-*nulls; they were statistically indistinguishable (Fig. 3C). To further control for variation in OV diameters between larvae, we measured the length of the *tbx1* domain (Fig. 3A) and expressed it as a fraction of the diameter of the same OV. In most Six1WT-injected *six1-*nulls, the *tbx1* domain appeared larger upon visual inspection (Fig. 4B; Table 2), which was confirmed by calculating the length/diameter ratio (L/D; Fig. 4C), indicating that Six1WT can restore *tbx1* OV expression.

**Table 2.** Percentages of embryos with the noted intensity of ISH staining scored as fainter, larger/darker or the same

	Nulls				Heterozygotes			
	Uninjected*	Six1WT	V17E	Y129C	Uninjected*	Six1WT	V17E	Y129C
*tbx1*	(14)	(11)	(18)	(22)	(36)	(18)	(35)	(45)
Smaller	**100**	0	16.7	50.0	33.0	44.4	25.7	33.3
Larger	0	63.6	5.6	9.1	0	16.7	2.9	11.1
Same	0	36.4	77.7	40.9	67.0	38.9	71.4	55.6
Larger+same	0	**100**	**83.3**	**50.0**	**67.0**	**55.6**	**74.3**	**66.7**
*dlx5*	(11)	(9)	(9)	(20)	(52)	(17)	(19)	(45)
Fainter	**100**	44.5	11.1	25.0	40.4	0	26.3	26.7
Darker	0	33.3	33.3	15.0	0	29.4	15.8	8.9
Same	0	22.2	55.6	60.0	59.6	70.6	57.9	64.4
Darker+same	0	**55.5**	**88.9**	**75.0**	**59.6**	**100**	**73.7**	**73.3**
*sobp*	(6)	(9)	(19)	(20)	(18)	(11)	(33)	(43)
Fainter	**100 ^‡^**	33.3 **^‡^**	78.9 **^‡^**	100 **^‡^**	11.1	27.3	21.2	16.3
Darker	0	66.7	21.1	0	0	18.1	12.1	7.0
Same	0	0	0	0	88.9	54.6	66.7	76.7
Darker+same	0	**66.7**	**21.1**	**0**	**88.9**	**72.7**	**78.8**	**83.7**

*For uninjected *six1-*nulls and uninjected *six1* heterozygotes, staining was compared to uninjected wild types.

^‡^For *sobp*-stained *six1*-nulls, ‘fainter’ means no stain detected.

For mRNA-injected embryos, staining on the injected side was compared to the uninjected side of the same embryo.

Bold indicates effects of: (1) in uninjected nulls/heterozygotes; (2) Six1WT differences from uninjected nulls/heterozygotes; and (3) corresponding effects cause by the variants. Numbers in parentheses indicate the number of embryos examined.

*dlx5* and *sobp* expression domains were not sufficiently discrete to measure, so instead the staining intensity on the injected side was visually scored as ‘fainter’, ‘darker’ or ‘same’ compared to the uninjected side of the same larva. In uninjected *six1*-nulls, *dlx5* OV expression was fainter compared to wild type (Fig. 4I). In comparison, many Six1WT-injected *six1-*nulls also had fainter *dlx5* staining (Fig. 4J), but the proportion was smaller and more larvae had darker or the same staining (Table 2; *P*>0.05). In uninjected *six1-*nulls, *sobp* expression was not detected, whereas it was strongly expressed in wild type (Fig. 4P). In comparison, the majority of Six1WT-injected *six1-*nulls had darker *sobp* staining on the injected side (Fig. 4Q; Table 2; *P*>0.05). For both *dlx5* and *sobp*, the OV diameters on the Six1WT-injected sides were indistinguishable from the uninjected sides (Fig. 4K,R), indicating that increased staining was not likely due to altered OV diameters. Thus, in *six1-*nulls Six1WT can partially restore both *dlx5* and *sobp* OV expression.

We next tested whether BOR variants restored OV gene expression as effectively as Six1WT. Notably, neither variant caused a significant change in OV diameter (Figs 3C, 4M,O,T,V), indicating that the expression differences described below are not likely due to variations in OV diameter. For *tbx1*, most V17E-injected *six1*-nulls had visually larger or same size domains (Fig. 4D; Table 2). The L/D indicated that in most embryos the *tbx1* domain was the same size on injected versus uninjected sides (Fig. 4E), which was significantly different from and thus less effective than Six1WT (Fig. 4H). The majority of Y129C-injected *six1*-nulls had visually smaller *tbx1* domains (Fig. 4F; Table 2) and a significantly smaller L/D (Fig. 4G). This also was different from Six1WT, i.e. was less effective, but did not differ from V17E (Fig. 4H). For *dlx5*, both V17E- and Y129C-injected *six1*-nulls had proportionally more larvae with darker (e.g. Fig. 4L) or the same (e.g. Fig. 4N) staining compared to Six1WT (Table 2), but these proportions were not significantly different from Six1WT or each other (*P*>0.05). In most V17E- and all Y129C-injected *six1*-nulls, *sobp* staining was undetectable (Fig. 4S,U; Table 2). Both variants were less able to restore *sobp* compared to Six1WT (V17E, *P*=0.0346; Y129C, *P*=0.0002); Y129C was significantly less effective compared to V17E (*P*=0.0471).

In summary, although BOR is a heterozygous condition, expressing Six1WT and variants in *six1*-null larvae revealed important differences between their effects: Six1WT partially restored *tbx1* and *sobp* OV expression, whereas both variants were less effective, Y129C significantly so. In contrast, both variants partially restored *dlx5* in proportions similar to Six1WT and to each other.

**Fig. 5.**
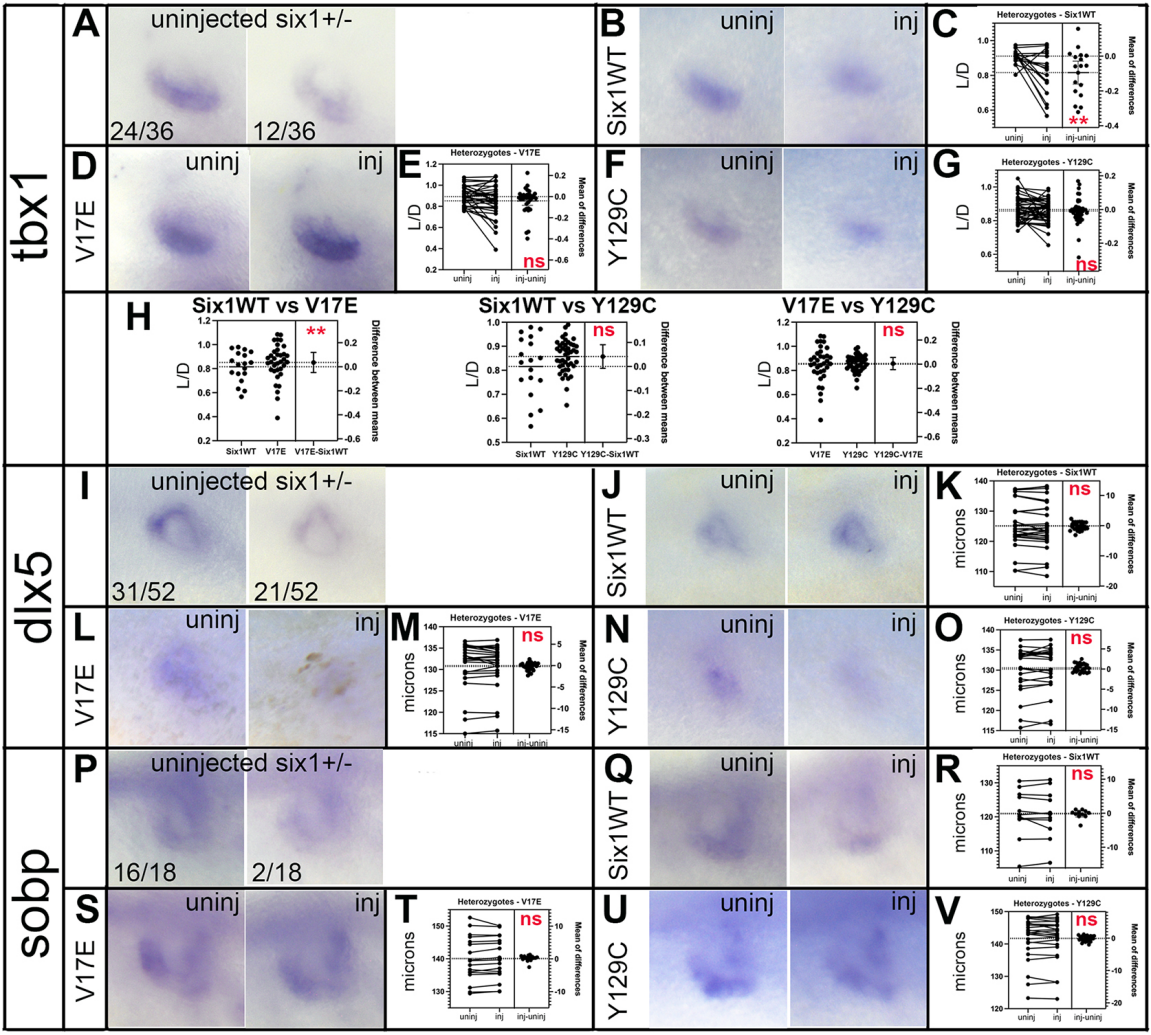
**In *six1* heterozygotes, BOR variants differentially alter OV gene expression.** (A) In uninjected *six* heterozygotes, one-third (12/36) of animals had fainter *tbx1* OV staining compared to uninjected wild types. (B) Most Six1WT-injected *six1* heterozygotes had a visually smaller *tbx1* domain on the injected (inj) versus uninjected (uninj) side of the same embryo. (C) The lengths of the *tbx1* domain/OV diameters (L/D) on each side of Six1WT-injected *six1* heterozygotes were significantly smaller. **P<0.01. (D) In most V17E-injected *six1* heterozygotes, the *tbx1* OV staining appeared the same on both sides. (E) The L/Ds on each side of V17E-injected *six1* heterozygotes were not significantly different. (F) In most Y129C-injected *six1* heterozygotes, the *tbx1* OV staining appeared smaller on the injected side. (G) The L/Ds on each side of Y129C-injected *six1* heterozygotes were not significantly different. (H) The LDs of Six1WT-injected versus V17E-injected *six1* heterozygotes were significantly different (**P<0.01). Y129C was not significantly different from Six1WT or V17E. (I) *dlx5* OV expression was fainter in 21/52 uninjected *six1* heterozygotes compared to wild types. (J.) In many Six1WT-injected *six1* heterozygotes, *dlx5* OV staining appeared darker on the injected side. (K) The OV diameter was not significantly different between injected and uninjected sides of Six1WT-injected *six1* heterozygotes stained for *dlx5*. (L) In many V17E-injected *six1* heterozygotes, *dlx5* OV staining was fainter on the injected side. (M) The OV diameter was not significantly different between injected and uninjected sides of V17E-injected *six1* heterozygotes stained for *dlx5*. (N) In some Y129C-injected *six1* heterozygotes, *dlx5* OV staining was fainter on the injected side. (O) The OV diameter was not significantly different between injected and uninjected sides of Y129C-injected *six1* heterozygotes stained for *dlx5*. (P) *sobp* OV staining was rarely fainter (2/18) in uninjected *six1* heterozygotes compared to wild types. (Q) A few Six1WT-injected *six1* heterozygotes had fainter *sobp* staining on the injected side. (R) The OV diameter was not significantly different between injected and uninjected sides of Six1WT-injected *six1* heterozygotes stained for *sobp*. (S) In most V17E-injected *six1* heterozygotes, *sobp* staining was the same on both sides. (T) The OV diameter was not significantly different between injected and uninjected sides of V17E-injected *six1* heterozygotes stained for *sobp*. (U) An example of fainter *sobp* staining on the injected side of a Y129C-injected *six1* heterozygote. (V) The OV diameter was not significantly different between injected and uninjected sides of Y129C-injected *six1* heterozygotes stained for *sobp*. ns, not significant. Error bars represent s.d. Dotted lines indicate 95% confidence interval.

To better model BOR, we next expressed these mRNAs in heterozygotes. On average, control heterozygote OV diameters were similar to those of wild types (Fig. 3B), and for each experimental group there were no significant differences between injected and uninjected OV diameters (Figs 3D and 5K,M,O,R,T,V), indicating that the differences in expression domains reported below are not likely due to changes in OV size. In uninjected heterozygotes, *tbx1* was expressed in all OVs, but in a visually smaller or fainter domain in one-third of animals (Fig. 5A; Table 2). Most Six1WT-injected heterozygotes also had a visually smaller domain and smaller L/D (Fig. 5B,C; Table 2). *dlx5* was expressed in the OV of all uninjected heterozygotes, but was fainter than wild-type staining in ∼40% (Fig. 5I; Table 2). In significant contrast, all Six1WT-injected heterozygotes had the same or darker *dlx5* OV staining (Fig. 5J; Table 2; *P*<0.0001). In uninjected heterozygotes, *sobp* was expressed in all OVs, and rarely was fainter (Fig. 5P; Table 2). In comparison, although proportionally more Six1WT-injected heterozygotes had fainter *sobp* OV staining (Fig. 5Q; Table 2), the difference was not significant (*P*>0.05). Thus, providing additional Six1WT to heterozygotes reduced *tbx1* domains, increased *dlx5* expression, and had minimal effects on *sobp*.

We next assessed whether the effects of the BOR variants in heterozygotes were similar to those of Six1WT. For *tbx1*, fewer V17E-injected heterozygotes had visually smaller and more had the same staining and L/Ds (Fig. 5D,E; Table 2), and L/Ds were significantly different from those of Six1WT-injected heterozygotes (Fig. 5H). In contrast, the effects of Y129C on OV staining intensity and L/D were not different from those of Six1WT (Fig. 5F-H; Table 2) or V17E (Fig. 5H). For *dlx5*, a larger proportion of both V17E- and Y129C-injected heterozygotes had fainter staining (Fig. 5L,N; Table 2), but only Y129C was significantly different from Six1WT (*P*=0.0104). For *sobp*, most V17E- and Y129C-injected heterozygotes had the same staining intensity on injected and uninjected sides (Fig. 4S,U; Table 2); for both, the distribution was indistinguishable from Six1WT or each other (*P*>0.05).

Expressing Six1WT and variants in *six1* heterozygotes revealed important differences compared to uninjected heterozygotes. Six1WT caused more variation in *tbx1* OV expression; V17E effects on *tbx1* OV expression were similar to uninjected heterozygotes, whereas Y129C effects were similar to Six1WT; Six1WT restored *dlx5* OV expression, whereas V17E was slightly less and Y129C was significantly less effective; and neither Six1WT, V17E nor Y129C significantly altered *sobp* OV expression.

### Craniofacial cartilages are differentially perturbed by BOR variants

**Fig. 6.**
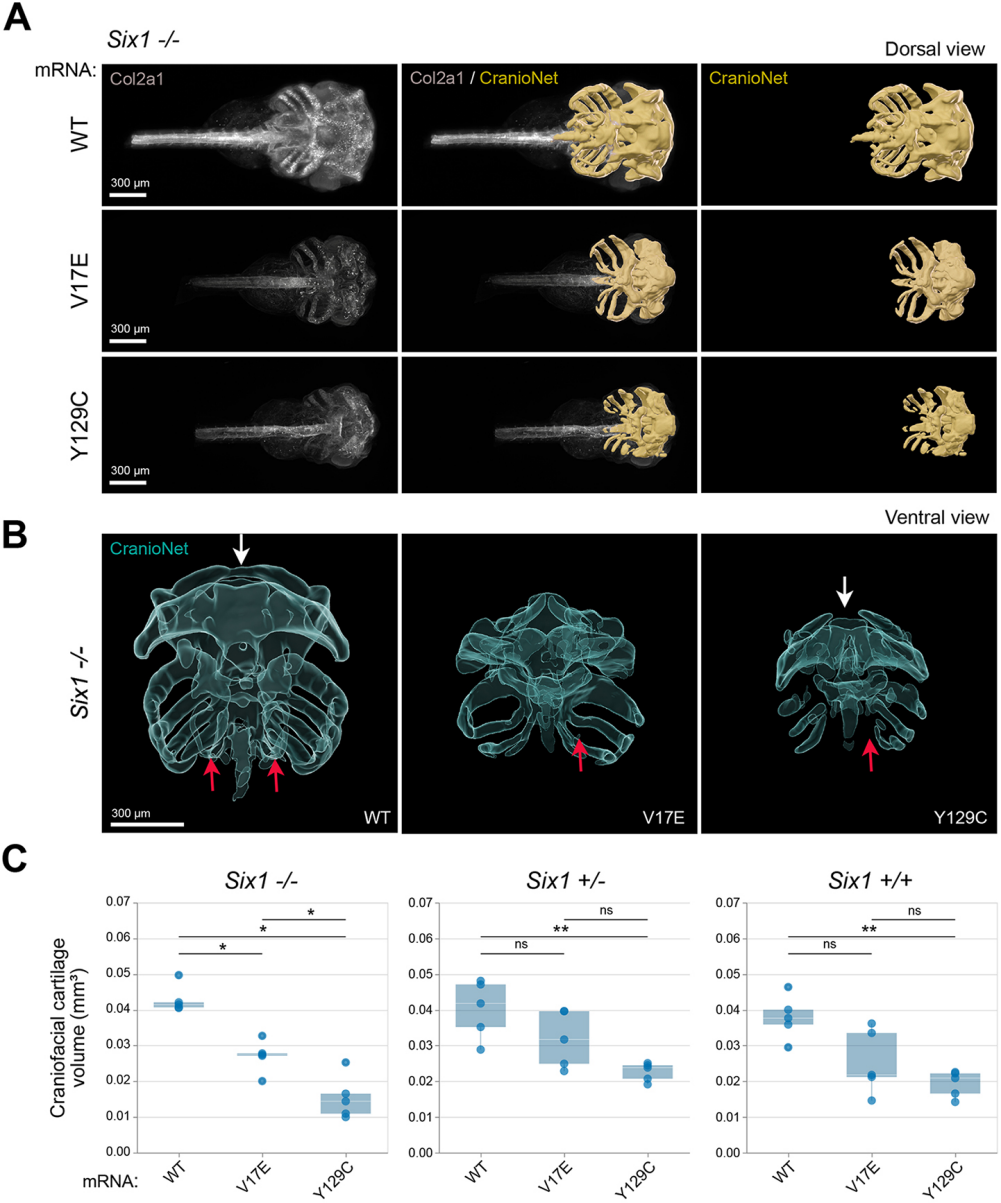
**Six1 BOR variants differentially rescue craniofacial cartilage formation.** (A,B) Representative 3D reconstructions of Col2a1-immunostained *six1*-null tadpoles injected with Six1WT, V17E or Y129C mRNA, imaged by mesoSPIM light-sheet microscopy and segmented using a finetuned CranioNet U-Net model. Injection of Six1WT mRNA restored cranial cartilage size without major defects, whereas V17E- and Y129C-injected tadpoles exhibited hypoplasia of multiple cartilaginous elements. (B) Most notable in the variant-injected tadpoles is agenesis of the infrarostral (white arrow) and reduced otic capsule (red arrows). (C) Quantification of total cranial cartilage volume. In *six1-null* tadpoles (left), Six1WT-injected animals had the largest volumes, which were reduced in V17E-injected and further decreased in Y129C-injected animals. Similar trends were observed in *six1^+/−^* (middle) and *six1^+/+^* (right) tadpoles. Box plots show the median (center line) and interquartile range (box limits, 25th and 75th percentiles); whiskers extend to the most extreme values within 1.5× the interquartile range, and individual points represent single tadpoles. Statistical tests were applied separately for each genetic background. For *six1*-nulls, data were not normally distributed (WT: Shapiro *P*=0.0091) and Mann–Whitney *U*-tests were used (WT versus V17E, *P*=0.0238; WT versus Y129C, *P*=0.0238; V17E versus Y129C, *P*=0.0476). For heterozygotes and wild types, normally distributed data were analyzed by two-sided, unpaired *t*-tests (WT versus V17E, ns; WT versus Y129C, *P*=0.0053 and *P*=0.0012, respectively). *P*-values were corrected for multiple comparisons using the Bonferroni method. *P<0.05, **P<0.01. ns, not significant.

In mouse and *X. tropicalis*, loss of Six1 results in hypoplastic otic and jaw cartilages (Laclef et al., 2003; Ozaki et al., 2004; Tavares et al., 2017; Coppenrath et al., 2021), as well as reduced cranial cartilage volume (Naert et al., 2021), tissues that are derived from the cranial NC. To determine whether Six1WT or the BOR-associated variants could rescue these defects, the respective mRNAs were injected into 1-cell stage *X. tropicalis* and cranial cartilages analyzed at tadpole stages (Fig. 1D). First, tadpoles were immunostained for Col2a1 and imaged using mesoSPIM light-sheet microscopy (Vladimirov et al., 2024). A base CranioNet U-Net model (Naert et al., 2021) was then fine-tuned for these recordings to segment and reconstruct the cartilaginous elements in 3D (Fig. 6A). Qualitatively, injection of Six1WT mRNA into *six1-*null tadpoles restored cranial cartilage morphology without discernible craniofacial defects. In contrast, V17E- and Y129C-injected *six1*-null tadpoles displayed gross malformations of cranial cartilages, most notably hypoplasia of the otic capsule (OC) and agenesis of the infrarostral (IF) cartilage (Fig. 6A,B). Quantitatively, *six1*-null tadpoles injected with Six1WT mRNA exhibited the largest cranial cartilage volume, which was reduced in V17E-injected animals (*P*<0.05) and further decreased in Y129C-injected animals (*P*<0.05) (Fig. 6C, left). To rule out overexpression effects, the same mRNAs were injected into *six1*-heterozygous and wild-type embryos (described below); in all backgrounds, Six1WT, V17E and Y129C mRNAs produced similar craniofacial abnormalities, indicating that the differences observed in *six1*-null tadpoles reflect variant-specific alterations in Six1 function rather than nonspecific dosage effects.

**Fig. 7.**
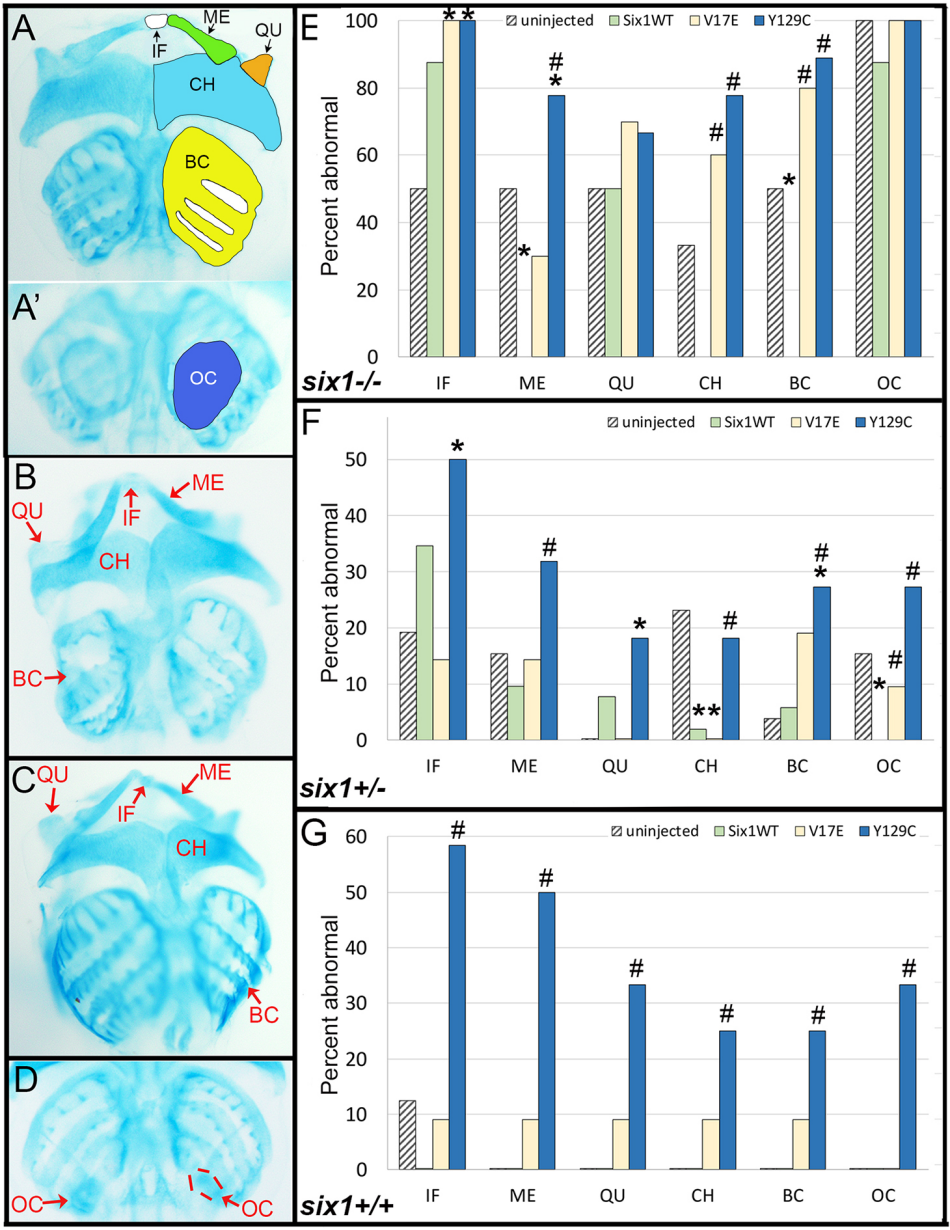
**The Y129C variant disrupts morphologies of craniofacial cartilage elements.** (A) Normal craniofacial cartilage morphology from a ventral view of an Alcian Blue-stained head of an uninjected *six1^+/+^* tadpole. The single infrarostral (IF, white) is located at the midline and articulates with Meckel's cartilage (ME, green). The quadrate (QU, orange) is triangular in shape, the ceratohyal (CH, light blue) is broad and gently curved and the branchial cartilage (BC, yellow) consists of four cartilaginous rows. (A′) From a dorsal view of the same tadpole, the oval otic cartilage (OC; dark blue) surrounds the inner ear tissues. (B-D) Examples of dysmorphic cranial cartilages (red arrows) in injected tadpoles. (B) In a V17E-injected *six1*-null, the IF is barely stained, indicating lack of cartilage differentiation, the MEs are concave and short, one QU is rounded, the CH are narrow, and one BC is small without regular rows. (C) In a V17E-injected heterozygote, the IF and one ME are short, the other ME has a crook, one QU is rounded and one BC is small. (D) In a Y129C-injected heterozygote, both OC are greatly reduced in size (compare to A′). (E) The percentage of *six1*-nulls showing cranial cartilage abnormalities similar to those illustrated in B-D, in uninjected tadpoles (hatched; *n*=14) and those injected with Six1WT (green; *n*=8), V17E (yellow; *n*=10) or Y129C (blue; *n*=9). (F) The percentage of *six1* heterozygotes showing abnormalities similar to those illustrated in B-D, in uninjected tadpoles (*n*=26) and those injected with Six1WT (*n*=52), V17E (*n*=21) or Y129C (*n*=22). (G) The percentage of *six1* wild types showing abnormalities similar to those illustrated in B-D, in uninjected tadpoles (*n*=8) and those injected with Six1WT (*n*=19), V17E (*n*=11) or Y129C (*n*=12). *P<0.05 compared to uninjected; **P<0.01 compared to uninjected; ^#^*P*<0.05 compared to Six1WT-injected.

To more specifically ascertain abnormalities in selected cartilages, tadpoles were stained with Alcian Blue, which allows analysis of individual cranial cartilages, and these elements were assessed for changes in staining compared to uninjected tadpoles (Fig. 7A). Elements were scored as abnormal if they were missing, smaller in size, misshaped, faintly stained or unstained (examples shown in Fig. 7B-D). Uninjected *six1*-null tadpoles frequently had missing IF and/or misshaped Meckel's (ME), quadrate (QU), ceratohyal (CH), branchial (BC) and otic capsule (OC) cartilages (*n*=14; Fig. 7E). In contrast, those injected with Six1WT (*n*=8) had normal ME, CH and BC (Fig. 7E). In addition, all contained IF, QU and OC, but IF and OC were only faintly stained and half of QU were rounded (Fig. 7E). Thus, providing Six1WT restored many cartilage elements. In comparison, the BOR variants were less effective. All *six1*-null tadpoles injected with V17E (*n*=10) had defective IF (30% missing, 50% unstained, 20% misshaped) and many had smaller or faintly stained ME (30% smaller), QU (60% smaller, 10% unstained), CH (60% smaller), BC (80% smaller) and OC (20% unstained, 80% smaller). For each element, V17E was less effective than Six1WT at restoring the cartilage, but the percentages of defects were significantly different only for CH (*P*<0.05) and BC (*P*<0.01). Similarly, all *six1*-null tadpoles injected with Y129C (*n*=9) had defective IF (33% missing, 45% unstained, 22% faint) and OC (11% unstained, 11% fainter, 78% smaller) and most had defective QU (56% smaller; 11% unstained). However, many more had smaller or misshaped ME (78%), CH (78%) and BC (89%). Like V17E, Y129C was less effective than Six1WT at restoring the cartilages, significantly for ME (*P*<0.005), CH (*P*<0.005) and BC (*P*<0.0001). These data indicate that, while Six1WT restored the morphology of many of the cartilage abnormalities observed in uninjected *six1*-null tadpoles, both variants were similarly unable to restore cartilage morphology in the absence of endogenous Six1.

Because individuals affected by BOR carry only one allele of a *SIX1* variant, we next analyzed the effects of the various mRNAs on cranial cartilage volume in heterozygous tadpoles (*six1^+/−^*). Compared to Six1WT-injected tadpoles, total cartilage volume was slightly reduced by V17E but significantly decreased by Y129C (*P*<0.01; Fig. 6C, middle). Analysis of Alcian Blue-stained tadpoles showed that in uninjected heterozygotes each cranial cartilage was abnormal in only a low percentage of tadpoles (∼20% or lower; Fig. 7F). Injection of Six1WT did not significantly change these percentages in most cartilages, but did restore the morphology of CH (*P*<0.005) and OC (*P*<0.01). V17E had similar effects as Six1WT, significantly differing only in its ability to restore OC (*P*<0.001). In contrast, Y129C caused a significant increase in defects in IF (*P*<0.05), QU (*P*<0.05) and BC (*P*<0.05) compared to uninjected heterozygotes, as well as a significant increase in defects in ME (*P*<0.05), CH (*P*<0.05), BC (*P*<0.02) and OC (*P*<0.0001) compared to Six1WT. Thus, defects were more frequent in heterozygotes expressing Y129C.

These results suggest that Y129C may interfere with the activity of residual endogenous Six1. To further assess this possibility, we injected the various mRNAs into wild-type embryos that carry normal levels of Six1. Here, we also found that the total cranial cartilage volume was slightly reduced in V17E-injected but significantly reduced in Y129C-injected tadpoles (*P*<0.01) compared to Six1WT (Fig. 6C, right). Analysis of Alcian Blue-stained tadpoles showed that Six1WT and V17E caused very few cartilage abnormalities, whereas Y129C caused defects at significantly higher frequencies (Fig. 7G). These data indicate that Y129C likely interferes with the function of endogenous Six1 during cranial cartilage development.

## DISCUSSION

Six1 plays a key transcriptional role in the development of the cranial sensory placodes, OVs and branchial arches. Heterozygous inheritance of *SIX1* variants in BOR result in highly variable levels of hearing loss and craniofacial dysmorphologies; however, it is not clinically obvious whether there are phenotypic differences between individuals carrying different variants (Smith and Azaiez, 2025). Because the clinical diagnostic criteria for BOR are the result of a multitude of developmental processes that cannot be interrogated after birth, we posited that exploring the effects of variants during embryogenesis would reveal underlying causes of those phenotypes and their variability. Therefore, we attempted to distinguish between the effects of two functionally distinct variants – V17E and Y129C – on the development of precursor populations that give rise to the craniofacial tissues affected in BOR.

### BOR variants differentially affect gene expression in craniofacial progenitor populations

The cranial NC that migrate into the branchial arches give rise to craniofacial skeletal elements, including jaws, middle ear ossicles and outer ear cartilages, whereas the PPE gives rise to the inner ear. Therefore, we first examined whether BOR variants differentially affect NC or PPE gene expression patterns in morphants in which endogenous Six1 was reduced. Previous work in embryos with wild-type Six1 levels reported that V17E caused effects very similar to additional Six1WT, whereas Y129C effects were much weaker (Shah et al., 2020; Mehdizadeh et al., 2021). Herein, we show that the effects of the variants are different in their ability to restore normal gene expression patterns in embryos with reduced Six1. While providing Six1WT to morphants partially restored the normal patterns of *foxd3*, *irx1* and *sox11* expression, V17E, which does not bind Eya1, was less effective in restoring *foxd3* and *irx1* and had a minimal effect on *sox11*. In comparison, Y129C, which has deficient DNA-binding capability, only weakly restored *foxd3* and *irx1*, but was more effective than either Six1WT or V17E at restoring *sox11*. These results demonstrate that SD and HD variants have different effects on the genes required for the early development of NC and PPE progenitor populations. It is tempting to posit that V17E is less effective than Six1WT because it is unable to function as a transcriptional activator due to loss of Eya1 binding (Ikeda et al., 2002; Li et al., 2003; Silver et al., 2003). This is consistent with the previous observation that *sox11* PPE expression is expanded by either expressing a Six1-activator construct or co-expressing Six1 and Eya1 (Brugmann et al., 2004). We predict that the opposite effects of Y129C on *sox11* expression result from its ability to bind endogenous Eya1, thus making this activating co-factor unavailable to any residual Six1 that might promote *sox11* expression. However, the effects of each protein on expression domains were variable, especially for *sox11*. It remains to be tested whether this variability is due to variable compensation by other genes, such as the highly related Six2, or reflect variable levels of remaining Six1 in the morphants, a possible limitation of this methodology.

We eliminated this potential source of variability in morphants by examining later stages in a mutant line that could be genotyped (see Materials and Methods). At these later stages, we assayed three genes encoding proteins known to be required for OV development. Tbx1 is required for the specification of auditory and vestibular precursor cells (Vitelli et al., 2003; Raft et al., 2004; Xu et al., 2007), Dlx5 plays an early role in the vestibular morphogenesis (Merlo et al., 2002), and Sobp regulates Six1 transcriptional activity during OV formation (Tavares et al., 2021). We found that in *six1*-nulls, Six1WT could partially restore the OV expression of all three genes, whereas V17E was less effective at restoring *tbx1* and *sobp*, and Y129C was less effective than V17E. Thus, clear differences between the BOR variants were revealed in *six1*-nulls. The BOR variants also had different effects in *six1* heterozygotes that best model BOR. For *tbx1*, Six1WT and Y129C had pleiotropic effects, whereas V17E effects were not different from uninjected *six1* heterozygotes. For *dlx5*, Six1WT restored expression, V17E was slightly less effective and Y129C was significantly less effective. For *sobp*, all three proteins caused pleiotropic effects that were not significantly different from uninjected *six1* heterozygotes or each other.

Thus, in both *six1* nulls and heterozygotes, we detected different effects of the BOR variants on OV gene expression, indicating that they act through distinct mechanisms. However, we have yet to elucidate the molecular mechanisms that cause these differences. While their different effects may be related to known differences in co-factor and DNA binding, during OV and branchial arch development there are complex regulatory interactions between Six1, Eya1, Tbx1, Dlx5 and Sobp (Freyer and Morrow, 2010; Guo et al., 2011; Li et al., 2020; Lin et al., 2009; Ozaki et al., 2004; Sato et al., 2010; Tavares et al., 2017, 2021; Zheng et al., 2003). Perhaps when levels of Six1 change or variants are introduced, these other genes or closely related Six family members compensate. Since inner ear development involves hundreds of genes (Chatterjee et al., 2010; Kolla et al., 2020; Baxi et al., 2023) and many additional Six1-binding partners (Neal et al., 2024), much experimentation will be needed to elucidate the precise molecular mechanisms that underlie the different effects of V17E and Y129C that we have uncovered.

### BOR variants have different effects on cranial cartilage morphology

Individuals with BOR present with abnormalities in structures derived from the NC that populate the second pharyngeal arch, including the hyoid region, middle ear and external ear. Hypoplasia of the mandible, derived from the NC populating the first pharyngeal arch, also are noted (Smith and Azaiez, 2025). Since Six1 plays a role in pharyngeal arch development (Xu et al., 2002; Guo et al., 2011; Tavares et al., 2017; Coppenrath et al., 2021), we also investigated whether the formation of the cranial cartilages were differentially affected by the BOR variants. Quantitatively, we found that providing Six1WT restored cranial cartilage volume in *six1*-nulls, whereas it remained significantly reduced in V17E- and Y129C-injected tadpoles. Y129C-injected *six1*-nulls also had a higher incidence of cranial cartilage malformations. In heterozygotes and wild types, only Y129C-injected tadpoles had reduced cartilage volume and significantly more malformations, suggesting that it interferes with the function of endogenous Six1. In comparison, the effects of V17E tended to be similar to those of Six1WT, particularly in heterozygotes and wild-type tadpoles, consistent with a report that another SD variant – R110W – causes milder phenotypes compared to Y129C (Ruf et al., 2004). These results demonstrate that the early alterations in NC gene expression are manifested in later disruptions in morphogenesis. Differential effects on the multiple developmental processes occurring between NC specification and cartilage formation are likely to contribute to the phenotypic variability seen in animal models and humans.

### Phenotype variability

Analyses of several BOR families including over 400 individuals clearly show considerable phenotypic variability within and across families harboring the same variants in *EYA1* and *SIX1* (Smith and Azaiez, 2025). It has long been a goal to explain the underlying mechanisms for this variability. Recent evidence indicates that *EYA1* variants are more frequently associated with branchial arch and external ear malformations compared to *SIX1* variants (Lee et al., 2023). However, among the reported *SIX1* variants there is no clear correlation between the variant and the spectrum of dysmorphologies. For example, both V17E and Y129C variants are characterized by hearing loss, hyoid fistulae and preauricular pits (Ruf et al., 2004; Kochhar et al., 2008). However, since biochemical studies in cell culture clearly show that SD variants have defective interactions with EYA1 and HD variants have reduced ability to promote transcriptional activation (Ruf et al., 2004; Patrick et al., 2009; Shah et al., 2020; Lee et al., 2023), we anticipated that studying these variants in embryos would reveal clear developmental differences. Indeed, we found that the expression levels of several genes in the precursor populations of structures affected in BOR – NC, PPE and OV – as well as cranial cartilage formation were differentially altered by V17E versus Y129C. These results show that the variants cause early developmental changes that impact later morphogenesis.

There also was notable variability in the effects of each variant even on defined genetic backgrounds. For example, there was variability in expression domain sizes (reduced in some embryos and enhanced in others) in *six1*-nulls (Fig. 2N,O; Fig. 4E,G) and heterozygotes (Fig. 5C,E,G). Perhaps this is not surprising since there are differences in the frequency of SD versus HD variants in individuals from different ethnic backgrounds (e.g. Lee et al., 2023; Cho et al., 2024), and variable dysmorphologies in *six1*-null mice carried on different genetic backgrounds (Zheng et al., 2003). It will be important to assess whether the outliers in our datasets are due to modifiers that impact the relative expression levels of the variants and wild-type Six1. Although variants are stable and expressed at similar levels to wild-type Six1 when transfected into cultured cells (Ruf et al., 2004; Patrick et al., 2009; Shah et al., 2020; Lee et al., 2023), this has not yet been established in the embryo environment. Furthermore, there is evidence that Eya1 levels are required for normal Six1 expression (Xu et al., 2002; Lee et al., 2023), which could be a significant factor in the phenotypes observed in heterozygotes. Finally, it is becoming clear that, in addition to Eya1, there are many other potential Six1-binding proteins that function during craniofacial development (Neal et al., 2024). It will be important to test these other potentially contributing factors in the presence of BOR variants to better understand the underlying causes of phenotypic variation in humans.

## MATERIALS AND METHODS

### Obtaining embryos and genotyping

*X. laevis* embryos were obtained by either gonadotropin-induced natural mating or by *in vitro* fertilization of wild-type, outbred adults, as previously described (Sive et al., 2000; Moody, 2000, 2018). Embryos were selected at the 2-cell stage if the first cleavage furrow bisected the lightly pigmented region of the animal hemisphere to accurately identify the dorsal-ventral and left-right axes (Klein, 1987).

*X. tropicalis* embryos were generated by hormone-induced *in vitro* fertilization of adult pairs of −28/+ (*Xtr.six1^em2Horb^*, RRID:NXR_3145) *six1* mutants (Coppenrath et al., 2021). Confirmed oocyte-positive females were given 20 U of Pregnant Mare Serum Gonadotropin (PMSG) (BioVender, RP17827210000) and 200 U of human chorionic gonadotropin (hCG) (BioVender, RP17825010) (Wlizla et al., 2018), whereas virgin females were given 10 U of PMSG and 100 U of hCG to potentially reduce instances of ovarian hyperstimulation syndrome (Green et al., 2007). Larvae and tadpoles were fixed at appropriate stages and genomic DNA extracted from individual tail clips using DNeasy Blood & Tissue Kit (QIAGEN, 69506). PCR amplification of the targeted region was carried out using the following primers: forward primer, 5′-CCATGTCTATGCTGCCTTCC-3′; reverse primer, 5′-CCCTCAGTTTCTCTGCTTCC-3′. PCR products were purified using the NucleoSpin PCR Clean-up procedure (Macherey-Nagel, 740609.250) and mutations were confirmed by sequencing.

### Microinjection

When selected 2-cell-stage *X. laevis* embryos reached the 4-cell stage, both the dorsal and ventral blastomeres on the left side were microinjected in their animal region with an equimolar mixture of two Six1 antisense MOs (9 ng total; Fig. 1B), according to standard methods (Moody, 2018). These MOs, one of which binds to the ATG start site region and the other to the upstream 5′UTR, were previously validated to be specific and effective (Brugmann et al., 2004; Sullivan et al., 2019). When a subset of morphants reached the 8-cell stage, their dorsal animal and ventral animal daughter cells, which are the major contributors to the cranial NC and pre-placodal ectoderm (Moody and Kline, 1990), were microinjected with 150 pg of mRNAs encoding either *X. laevis* Six1WT, V17E or Y129C mixed with 100 pg of nβgal mRNA as a lineage tracer, as previously described (Shah et al., 2020). Both injections were done on only the left side of the embryo and the uninjected right side was used as an internal control to compare the sizes of the gene expression domains. Bilateral comparison within the same embryo is an essential control because there is up to a 38% variation in diameter and 2.6-fold difference in volume in *Xenopus* embryos, even in clutches derived from the same female (Leibovich et al., 2020). These size differences, as well as differences in embryo growth, result in variations in gene expression levels and domain sizes between embryos. Embryos were cultured in a diluted series of Steinberg's solution until fixation for ISH.

*X. tropicalis* embryos were microinjected at the 1-cell stage (for cartilage analyses) or into the animal region of a single blastomere at the 2-cell stage (for gene expression analyses) with 60 pg of either Six1WT, V17E or Y129C mRNAs mixed with Texas Red-dextran as a lineage marker (Thermo Fisher Scientific, D1829). Since *X. laevis* and *X. tropicalis* Six1 have identical amino acid sequences, with the exception of three conservative and one semi-conservative substitutions in the C-terminus (Fig. S2), the *X. laevis* protein is expected to be functionally identical when expressed in *X. tropicalis*. Microinjections performed at the 2-cell stage were targeted to one blastomere at random and screened at early tailbud stages for the fluorescent lineage marker to determine which side was injected. The opposite, uninjected side of the embryo served as an internal control for the ISH analyses. All embryos were reared according to Wlizla et al. (2018) until collected for fixation (Fig. 1C,D).

### ISH

*X. laevis* embryos were cultured to neural plate stages (16-18) and *X. tropicalis* to larval stages (28-32) (Nieuwkoop and Faber, 1994; Zahn et al., 2022), fixed in 4% paraformaldehyde (in 0.1 M MOPS, 2 mM EGTA Magnesium, 1 mM MgSO_4_, pH 7.4) and processed for ISH as described previously (Sive et al., 2000). Digoxigenin-labeled antisense RNA probes (*dlx5*, *foxd3*, *irx1*, *sobp*, *sox11*, *tbx1*) were synthesized *in vitro* (MEGAscript kit; Thermo Fisher Scientific) from cDNA plasmids as previously described (Sive et al., 2000). The expression patterns were compared between the injected and control sides of the same embryo and visually scored as follows. For neural plate stage embryos, ‘fainter’ means fainter intensity (e.g. Fig. 2C,D); ‘larger’ means same intensity but bigger size (e.g. Fig. 2B,J); and ‘same’ means both same intensity and same size (e.g. Fig. 2M). The proportions of embryos in each category were compared between experimental groups by a two-sided Fisher's exact test (GraphPad Prism 11). In a subset of these embryos, the perimeter of the expression domain was measured on both the injected and uninjected sides of the same embryo using the measuring tool in the cellSens Entry software (Olympus/Evident Scientific) (Fig. S1). Sizes between injected and uninjected sides were compared by paired, two-tailed *t*-tests (GraphPad Prism 11; e.g. Fig. 2N,O,P). For OV-stage larvae, ‘fainter’ means fainter intensity (e.g. Fig. 4J); ‘smaller’ means shorter length (e.g. Fig. 4G); ‘larger’ means same intensity but bigger size (e.g. Fig. 4B); and ‘same’ means both same intensity and same size (e.g. Fig. 4D). The proportions of embryos in each category were compared between experimental groups by a two-sided Fisher's exact test (GraphPad Prism 11). In all larvae, the diameter of the OV in the dorsal-ventral axis was measured using the measuring tool in the cellSens Entry software (Olympus/Evident Scientific) (Fig. 3A). For larvae stained for *tbx1*, the length of the expression domain was measured on both the injected and uninjected sides of the same embryo and expressed as a percentage of the OV on each side (L/D) (Fig. 3A). Sizes of injected and uninjected sides of the same embryo were compared by paired, two-tailed *t*-tests and those between experimental groups were compared by unpaired, two-tailed *t*-tests (GraphPad Prism 11). Whole-mount ISH images were collected with an Olympus SZX16 stereomicroscope coupled to an Olympus UC90 camera.

### Tadpole light-sheet microscopy and CranioNet segmentation

Whole-mount Col2a1 immunostaining of *X. tropicalis* tadpoles was performed as described previously (Naert et al., 2021). Stained specimens were imaged using mesoSPIM light-sheet microscopy at either 5× or 10× magnification (Voigt et al., 2019; Vladimirov et al., 2024). For volumetric normalization across magnifications, reconstructed image stacks were scaled using a cubic correction factor derived from the notochord diameter.

A base 2D CranioNet U-Net model (Naert et al., 2021) was fine-tuned for these recordings to segment and reconstruct cartilaginous elements. For training, every 20th optical section was exported from one representative *z*-stack per genotype and injection condition using a Fiji macro. The resulting images from all conditions were pooled, and approximately 25% of slices were randomly assigned to the validation dataset using an automated file selection script, ensuring balanced representation across genotypes (*six1*^+/+^, *six1*^+/−^, *six1*^–/–^) and injection conditions (Six1WT, V17E, Y129C). In total, 66 images were used for training and 22 for validation. The CranioNet model was fine-tuned for 20,000 iterations at a learning rate of 1×10^−4^, followed by 2000 iterations at 2×10^−5^, reaching an intersection-over-union (IoU) of 0.57 on the validation set.

The resulting segmentation data were further processed using Imaris (Bitplane) for 3D visualization and volumetric quantification. Cranial cartilage and notochord diameters were measured using Imaris measurement tools, and all 3D renderings shown in figures were generated in Imaris. Statistical analysis and data visualization were performed in Python using ‘pandas’, ‘numpy’, ‘scipy.stats’ and ‘altair’.

### Alcian Blue staining

Alcian Blue staining of *X. tropicalis* tadpole cranial cartilage was performed according to Young et al. (2017). Fixed and genotyped tadpoles were incubated in a solution of acid/alcohol containing 0.1% Alcian Blue. When staining was complete, tadpoles were washed in the acid/alcohol solution without Alcian Blue, bleached with a solution containing 1.2% hydrogen peroxide and 5% formamide and cleared in 2% KOH with increasing concentrations of glycerol. The frequency of morphological defects between groups were compared by a two-sided Fisher's exact test (GraphPad Prism 11). Whole-mount images were captured using an Olympus SZH16 stereomicroscope coupled with an Olympus UC90 camera and cellSens Entry software.

## Supplementary Material

10.1242/develop.205428_sup1 Supplementary information
