# Supplementary material for: SIX1 branchio-oto-renal syndrome variants have different effects on embryonic craniofacial gene expression and cartilage formation
Source: Development. 2026 Jul 8;153(13):dev205428. doi: 10.1242/dev.205428 (PMC13347299; doi:10.1242/dev.205428)
Supplement: Supplementary information [file develop-153-205428-s1.pdf]

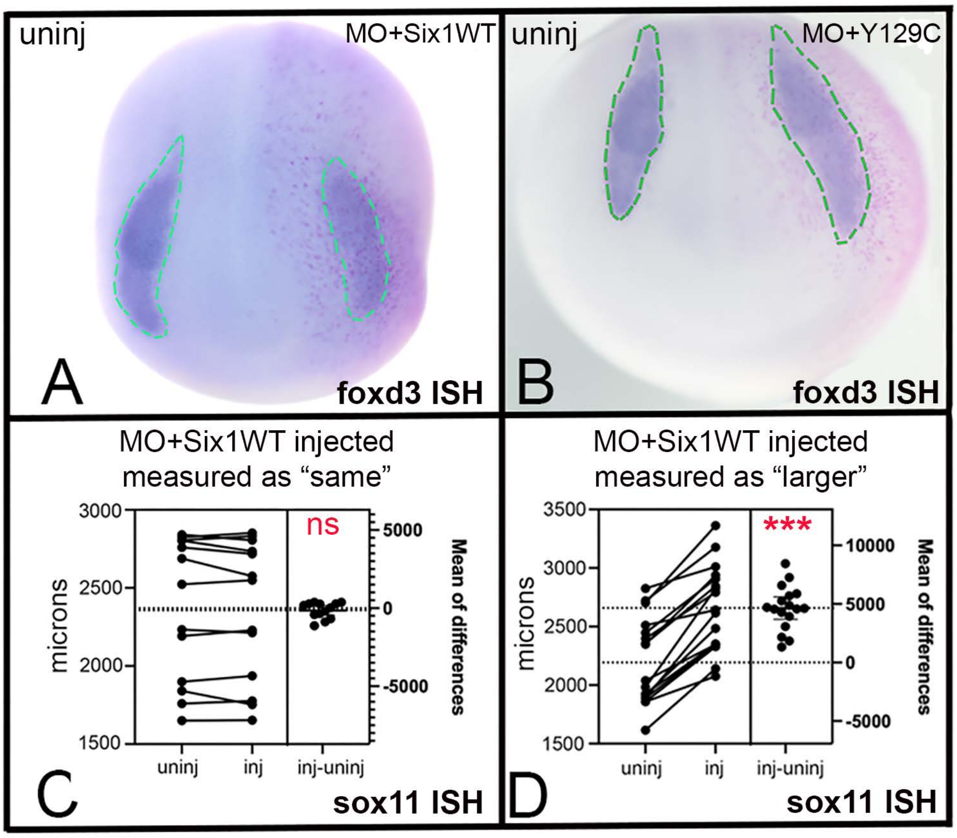

Fig. S1. Measuring expression domain sizes in morphants

A. An example outlining the *foxd3* expression domain (green dashes) on the MO+Six1WT-injected side (pink lineage dots) and control, uninjected side (uninj). Anterior view, dorsal to the top.
B. An example outlining the *foxd3* expression domain (green dashes) on the MO+Y129C-injected side (pink lineage dots) and control, uninjected side (uninj). Anterior view, dorsal to the top.
C. The sizes of the *sox11* domain in Six1WT-injected morphants, measured as in A, revealed that those scored as visually "same" showed no difference in size between uninjected (uninj) and injected (inj) sides of the same embryo. ns, $p>0.05$.
D. The sizes of the *sox11* domain in Six1WT-injected morphants, measured as in A, revealed that those visually scored as "larger", the size on the injected side (inj) was significantly larger than on the uninjected side (uninj). ***, $p<0.001$.

Development • Supplementary information

```
NM_001100223 X.tropicalis: MSMLPSFGFTQEQVACVCEVLQQGGNLERLGRFLWSLPACDHLHKNESVLKAKAVVA
NM_001088558 X. laevis:    MSMLPSFGFTQEQVACVCEVLQQGGNLERLGRFLWSLPACDHLHKNESVLKAKAVVA
NM_005982.4 Homo sapiens:  MSMLPSFGFTQEQVACVCEVLQQGGNLERLGRFLWSLPACDHLHKNESVLKAKAVVA

FHRGNFRELYKILESHQFSPHNHPKLQQLWLKAHYVEAEKLRGRPLGAVGKYRVRRKFPLPRTIWDGEETSYCFKEKSRGVLREW
FHRGNFRELYKILESHQFSPHNHPKLQQLWLKAHYVEAEKLRGRPLGAVGKYRVRRKFPLPRTIWDGEETSYCFKEKSRGVLREW
FHRGNFRELYKILESHQFSPHNHPKLQQLWLKAHYVEAEKLRGRPLGAVGKYRVRRKFPLPRTIWDGEETSYCFKEKSRGVLREW

YAHNPYPSPREKRELAEATGLTTTQVSNWFKNRRQRDRAAEAKERENTENNNTSSNKQNQLSPLDGGKSLMSSSEEEFSPPQSPD
YAHNPYPSPREKRELAEATGLTTTQVSNWFKNRRQRDRAAEAKERENTENNNTSTNKQNQLSPLDGGKSLMSSSEEEFSPPQSPD
YAHNPYPSPREKRELAEATGLTTTQVSNWFKNRRQRDRAAEAKERENTENNNSSSNKQNQLSPLEGGKPLMSSSEEEFSPPQSPD

QNSVLLLQGSLTHPGGSSYSLSALSASQGGHGLQGHQHQLQDSLLGPLTSSLVDLGS
QNSVLLLQGSLTHPGATSYSLSALSASQGSHGLQGHQHQLQDSLLGPLTSSLVDLGS
QNSVLLLQGNMGHARSSNYSLPGLTASQPSHGLQTHQHQLQDSLLGPLTSSLVDLGS
```

Fig. S2. Comparison of the amino acid sequence of Six1 in *X. tropicalis* (black), *X. laevis* (red) and *Homo sapiens* (blue).

Sequence is identical between both *Xenopus* species and human in the protein-protein interaction domain (Six1 domain, underlined) and the homeodomain (bold). In the C-terminus of the protein, there are three conservative (yellow: S-T, G-A) and one semi-conservative (blue: G-S) amino acid substitutions in *X. laevis* compared to *X. tropicalis*. There are seven conservative substitutions (yellow: T-S, S-N, L-M, A-G, S-T), two semi-conservative (blue: G-S, A-S) and seven non-conservative (green: S-P, T-G, P-A, G-R, G-P, G-T) amino acid substitutions in the C-terminus of human compared to *Xenopus*.

Development • Supplementary information
